# Early Levodopa Therapy in Tyrosine Hydroxylase Deficiency

**DOI:** 10.1002/mdc3.70202

**Published:** 2025-07-01

**Authors:** Claudio M. de Gusmao, Roser Pons, Ganeshwaran H. Mochida, Laura Silveira‐Moriyama, Fernando Kok

**Affiliations:** ^1^ Department of Pediatric Neurology University of Sao Paulo Sao Paulo Brazil; ^2^ Department of Neurology Universidade Estadual de Campinas (UNICAMP) Campinas Brazil; ^3^ First Department of Pediatrics Aghia Sofia Hospital, National and Kapodistrian University of Athens Athens Greece; ^4^ Division of Genetics and Genomics and The Manton Center for Orphan Disease Research Boston Children's Hospital, Department of Pediatrics, Harvard Medical School Boston MA USA; ^5^ Pediatric Neurology Unit, Department of Neurology Massachusetts General Hospital Boston MA USA; ^6^ Teaching Unit, Institute of Neurology UCL Queen Square London United Kingdom

**Keywords:** genetics, levodopa, neurotransmitter disorders, tyrosine hydroxylase

Tyrosine hydroxylase (TH) deficiency (OMIM # 605407) is a neurotransmitter disorder caused by biallelic pathogenic variants in the *TH* gene, leading to deficits in dopamine and norepinephrine synthesis. Symptoms present in a spectrum that ranges from childhood‐onset dystonia to severe infantile parkinsonism‐dystonia.^1^ Treatment with levodopa is beneficial, but some individuals have diminished response and/or intolerable dyskinesias.^2^, ^3^ We report outcomes in 3 individuals with TH deficiency and review symptoms in a patient series based on the age of initiation of levodopa. Our findings suggest that early initiation of levodopa influences outcome and may prevent disease manifestations.

## Case Report

### Family 1

Sibling 1 was born after an uncomplicated pregnancy and labor. At 3 months of age, the family noted lower limb tremor. Shortly, tremor progressed to the upper extremities and tongue. Axial hypotonia, facial hypomimia, and oculogyric crises ensued. By 6 months of age, limb dystonia was present. Dystonia generalized and spasticity ensued, rendering the patient bed‐bound. At 13 years of age, he was diagnosed with TH deficiency using whole exome sequencing (pathogenic variants in *TH* NM_199292.3: c.698G>A; p.Arg233His and c.721G>A; p.Ala241Thr). He was started on levodopa, which improved oculogyric crises, hypomimia, and swallowing. Dystonia did not abate; small dosage increases led to severe dyskinesias. On current examination at 34 years of age, he can speak ~10 words and has limited verbal comprehension. There is generalized dystonia and spasticity, with contractures in both upper and lower extremities. Reflexes are brisk throughout. He cannot tolerate levodopa dosages higher than 0.2 mg/kg/day.

Sibling 2 is a female who first presented at 3 months with limb tremor, with subsequent motor delay and ptosis. TH deficiency was confirmed at 7 months of age with the identification of the same genotype as her sibling; levodopa was started immediately. There was gradual improvement in tone and development. At present, she is 21 years old, sociable, and with good performance in college. There is slight bradykinesia but otherwise no dystonia, pyramidal signs, or other findings. With higher dosages of levodopa, there is an inner sensation of unrest but no frank dyskinesias (Video 1).

**VIDEO 1:** First segment: sibling 1, age 34, is bed‐bound, with joint contractures, generalized body hypokinesia, dystonic postures, and hyperreflexia. Second segment: sibling 2, age 21. There is a slight decrement on repeated hand opening in the left upper extremity. No dystonic postures were seen. Gait is normal. Sibling 1 is individual 9 and sibling 2 is individual 3 in the table.

### Family 2

This boy was born after an uneventful pregnancy and labor. At 6 months of age, there was hand tremor, evolving to fisting and myoclonic jerks. Motor delay, ptosis, and oculogyric crises ensued. Exome sequencing demonstrated homozygosity for the *TH* pathogenic variant c.721G>A; p.Ala241Thr. He began levodopa at 2 years of age. On current examination at 13 years of age, he has mild autism and learning difficulties. Motorically, he is described as clumsy, but there is no parkinsonism, dystonia, or other abnormalities. Mild levodopa‐induced finger dyskinesias are noted (Video 2).

**VIDEO 2:** Patient from family 2, currently 11 years old. There is motor impersistence with mild dyskinesias in the upper limbs, with slight decrement on repetitive tasks without overt bradykinesia. Clumsiness is not accompanied by signs of ataxia. This is individual 5 in the table.

## Discussion

Clinical manifestations in TH deficiency vary in severity and include both motor and nonmotor symptoms. Treatment with levodopa may not lead to complete symptom resolution and is often challenging due to troublesome dyskinesias.^2^, ^3^ Previously, a classification proposed that individuals could be grouped into an infantile hypokinetic‐rigid syndrome (type A) or a complex encephalopathy (type B).^4^ More current literature suggests that TH deficiency occurs within a phenotypic spectrum. In fact, despite sharing the same genotype, the siblings described here were previously reported and grouped as type B (sibling 1) and type A (sibling 2).^4^

We hypothesize that early treatment leads to better outcomes. In sibling 2, starting levodopa at 5 months of age led to sustained improvement in motor deficits and normal intellect in adulthood. In contrast, sibling 1 who started treatment at 13 years of age has intellectual disability, dystonia, and spasticity. The patient from family 2 was started on levodopa at age 2. He had good motor response but remains with residual learning difficulties. Furthermore, follow‐up experience from one of the authors with 6 patients carrying the homozygous c.707T>C; p.Leu236Pro variant^3^ indicates that all those who began treatment within the first 6 months of life had good cognitive and motor outcomes. Table 1 presents clinical outcomes for all individuals, ordered based on the age of starting levodopa treatment. The therapeutic outcome appears multidimensional, expanding from motor to nonmotor symptoms. All individuals who started levodopa after age 2 had some degree of cognitive impairment, with particularly poor motor and cognitive outcomes for those who started after age 8.

**TABLE 1:** Clinical symptoms and outcomes of individuals with tyrosine hydroxylase (TH) deficiency based on the age of starting levodopa

	Presentation					Levodopa						Follow‐up			
	Disease onset	Other symptoms	Genotype^a^	Brain MRI	Prolactin (ng/dL)	Start	Response	Side effects	Current dose	Maximum dose	Interval	L‐dopa‐induced dyskinesias?	Age	ID	Other findings
1	3 mo, OGC	Motor delay, tremor (tongue and limbs)	c.707T>C; c.707T>C	Normal	25.4	5 mo	↓ OGC, gradual motor progress	Dyskinesias	5.7 mg/kg/d	11 mg/k/d	TID	Yes, at 1 mg/k/d	17 y	No	Mild finger dyskinesias
2	2 mo, OGC, tremor, motor delay		c.707T>C; c.707T>C	Pituitary changes	47.1	6 mo	↓ OGC, gradual motor progress	Dyskinesias	6.8 mg/kg/d	9 mg/k/d	TID	Yes, at 0.5 mg/k/d	14 y	No	Mild finger dyskinesias
3	3 mo, tremor in legs	Ptosis, OGC, lingual tremor	c.698G>A; c.721G>A	Normal	11	7 mo	Immediate response + gradual motor progress	None	5 mg/kg/d	current	QID	No	21 y	No	Slight asymmetric bradykinesia
4	6 mo, hypotonia, dev. delay	OGC	c.707T>C; c.707T>C	Normal	5.1	11 mo	↓ OGC, gradual motor progress	Dyskinesias	7 mg/kg/d	12 mg/k/d	TID	Yes, at 1.5 mg/k/d	7 y	No	Normal
5	6 mo, tremor in upper limbs	Ptosis, OGC, dystonic postures	c.721G>A; c.721G>A	n/a	36	23 mo	Walked within weeks	None	2.8 mg/kg/d	Current	TID	Yes, at 2.8 mg/kg/d	13 y	Mild	ASD, learning difficulties. Mild dyskinesias.
6	3 mo, tremor in four limbs	OGC, motor delay	c.707T>C; c.707T>C	Pituitary changes	161	24 mo	↓ OGC, gradual motor progress	Dyskinesias	5.7 mg/kg/d	7 m/k/d	TID	Yes, at 2 mg/k/d	19 y	Yes	ID, dysarthria, facial and upper limb dyskinesias
7	5 mo, tremor in four limbs	OGC, motor delay	c.707T>C; c.707T>C	Normal	49.6	25 mo	↓ OGC, gradual motor progress	Dyskinesias, nausea	3.3 mg/kg/d	6 mg/k/d	TID	Yes, at 5 mg/k/d	18 y	Yes	Dysarthria, bradykinesia, facial and upper limb dyskinesias.
8	4 mo, tremor, dystonia, dev. delay	OGC	c.707T>C; c.707T>C	Normal	n/a	8 y	None	Dyskinesias	n/a	4 mg/k/d	TID	Yes, at 3.3 mg/k/d	22 y	Yes	Generalized dystonia, multiple contractures
9	3 mo, tremor in legs	OGC, dystonic posturing, lingual tremor	c.698G>A; c.721G>A	Normal	n/a	13 y	↓ OGC, slightly improved swallowing	Dyskinesias	0.2 mg/kg/d	2 mg/kg/d	QD‐BID	Yes, >0.2 mg/kg/d	34 y	Yes	Generalized dystonia, spasticity and contractures.

^a^ Reference transcript NM_199292; respective protein changes by mutation: c.698G>A‐p.Arg233His; c.721G>A‐p.Ala241Thr; c.707T>C‐p.Leu236Pro. Patients 1, 2, 6, 7, and 8 were previously reported in Pons et al., 2013. Patients 3 and 9 are the siblings of Video 1 and were previously reported in Wilemsen et al., 2010. Patients 4 and 5 (Video 2) are newly reported in this publication.

Abbreviations: OCG, oculogyric crisis; ↓ OGC, decreased frequency of OGCs; TID, ter in die; QID, quater in die; ASD, autism spectrum disorder; ID, intellectual deficiency; dev. delay, developmental delay; BID, bis in die.

In patients with TH deficiency, the enzymatic synthetic capacity is related to symptom severity.^5^ The variants reported here have a range of residual enzyme activity (p.Arg233His 14%; p.Ala241Thr<5%; p.Leu236Pro 16%),^6^ and all individuals had the commonality of better outcomes with early treatment. This suggests that age at levodopa initiation influences prognosis independently from residual enzyme activity. A review of cases reported in the literature endorses this hypothesis. A boy with the homozygous null mutation c.457C>T; p.Arg153* had better outcomes with levodopa started at 20 months of age when compared to individuals with residual activity but who started later^7^; siblings with TH deficiency also exhibit better results based on age of levodopa initiation.^5^, ^8^ The positive influence of early treatment is also supported by data from other hypodopaminergic states, such as deficits in tetrahydrobiopterin, in which early treatment is recommended.^9^

It is possible that other factors, such as polymorphisms in *TH*, modifier genes, or the environment also influence outcome. In one report, there was good motoric response despite delayed levodopa initiation.^10^ Further research is necessary to identify additional factors influencing therapeutic response in TH deficiency. Nevertheless, our experience suggests that age at treatment initiation is a strong predictor of outcome in at least a subset of individuals. Although the relationship between early initiation of levodopa and outcomes needs to be further prospectively investigated, we pose an argument for including TH deficiency in newborn screening.

## Author Roles

(1) Research project: A. Conception, B. Organization, C. Execution; (2) Statistical analysis: A. Design, B. Execution, C. Review and critique; (3) Manuscript preparation: A. Writing of the first draft, B. Review and critique; (4) Claudio M. de Gusmao.

C.M.d.G: 1A, 1B, 1C, 3A

R.P: 1A, 3B

G.H.M: 1B, 1C, 3B

L.S.‐M: 1B, 3B

F.K: 1A, 1C, 3B

## Disclosures

**Ethical Compliance Statement:** This study did not require an IRB approval. Patient assent was obtained verbally from individuals in families 1 and 2. Formal written consent was signed by the legal guardians of sibling 1 in family 1 and from the patient's legal guardians in family 2. Informed consent was directly obtained and signed by sibling 2 from family 1. We confirm that we have read the journal's position on issues involved in ethical publication and affirm that this work is consistent with those guidelines.

**Funding Sources and Conflict of Interest:** No specific funding was received for this work. All the authors declare that there are no conflicts of interest relevant to this work.

**Financial Disclosures for the Previous 12 Months:** Claudio M. de Gusmao was previously an employee of Mendelics Genomic analysis from 2021 to 2024; he has received small honoraria from PTC therapeutics and International Parkinson and Movement Disorders Society.

Roser Pons has served as a consultant for neurocrine; she has been paid by Genesis Pharma, Ardius, and AstraZeneca to participate in national and international congresses, and has participated in advisory boards organized by Novartis, Ardius, Biomarin, Genesis Pharma, and Roche.

Ganesh H. Mochida has received research support from NIH grant R21NS139341, unrelated to this current work. Laura Silveira‐Moriyama is currently an employee of Universidade Estadual de Campinas (UNICAMP), has received research support from Samsung Electronics and SAS Brasil, and small grants/honoraria from Ipsen, FQM‐Roche, AbbVie, Zambon, Merz, and International Parkinson and Movement Disorders Society. Fernando Kok is an employee of Mendelics Genomic Analysis and University of Sao Paulo.

## Data Availability

The data that support the findings of this study are available from the corresponding author upon reasonable request.
